# Naturally cultured high resistant starch rice improved postprandial glucose levels in patients with type 2 diabetes: A randomized, double-blinded, controlled trial

**DOI:** 10.3389/fnut.2022.1019868

**Published:** 2022-12-21

**Authors:** Ling-li Tan, Wei-qian Duan, Meng-xue Chen, Ying Mei, Xiao-ya Qi, Yong Zhang

**Affiliations:** ^1^School of Public Health and Health Management, Chongqing Medical University, Chongqing, China; ^2^Department of Health Management, The Second Hospital Affiliated to Chongqing Medical University, Chongqing, China

**Keywords:** resistant starch (RS), rice, diabetes, postprandial glucose level, randomized controlled trial (RCT)

## Abstract

**Objective:**

To assess the effect of a novel naturally cultured rice with high resistant starch (RS) on postprandial glycemia in patients with type 2 diabetes compared to ordinary rice.

**Design:**

This study is a randomized, double-blinded controlled trial.

**Methods:**

Patients with type 2 diabetes were recruited, and postprandial glucose levels were measured at 5-time points after the ingestion of one of two types of cooked rice in random order. Paired *t*-tests were used to compare postprandial blood glucose changes and increment areas under the blood glucose curve between high-RS rice and ordinary rice.

**Results:**

The increments of the postprandial blood glucose levels for high-RS rice were significantly lower than that for ordinary rice, i.e., 2.80 ± 1.38 mmol/L vs. 3.04 ± 1.50 mmol/L (*P* = 0.043) and 3.94 ± 2.25 mmol/L vs. 4.25 ± 2.29 mmol/L (*P* = 0.036) at 30 min and 60 min, respectively. The incremental areas under the blood glucose curve for high-RS rice were also significantly lower than that for ordinary rice, i.e., 42.04 ± 20.65 [mmol/(L·min)] vs. 45.53 ± 22.45 [mmol/(L·min)] (*P* = 0.043), 143.54 ±69.63 [mmol/(L·min)] vs. 155.15 ± 73.53 [mmol/(L·min)] (*P* = 0.026), and 354.61 ± 191.96 [mmol/(L·min)] vs. 379.78 ± 195.30 [mmol/(L·min)] (*P* = 0.042) at 30, 60, and 120 min, respectively. Repeated-measures ANOVA showed that postprandial glucose levels were not affected by the test order.

**Conclusion:**

The novel high-RS rice as a staple food when substituting for widely consumed ordinary rice may provide potential health benefits by lowering blood glucose in patients with type 2 diabetes.

## Introduction

Diabetes is one of the most common chronic non-communicable diseases in China and worldwide and has become a great public concern. According to the data released by the International Diabetes Federation in 2019, the global prevalence of diabetes was 9.3% (1). While the number of people with diabetes in China has exceeded 100 million, it is ranking first in the world (2). Most cases of diabetes worldwide are type 2 diabetes (3). The epidemic of type 2 diabetes affects patients' health to varying degrees and may even threaten their lives in serious cases (4). In addition, the high cost of treatment imposes a huge economic burden on individuals and countries (2). Currently, lifestyle interventions are the major preventive and basic therapeutic measures for patients with diabetes (5, 6). The main strategy for reducing the risk of complications and improving the quality of life of patients with diabetes is intensive glycemic control (7, 8). Therefore, dietary interventions as a part of lifestyle change play an important role in controlling blood glucose levels (9).

Starch is one of the main forms of carbohydrates in the diet and is classified into three categories according to its digestibility: rapidly digestible starch (RDS), slowly digestible starch (SDS), and resistant starch (RS) (10). RS cannot be digested and absorbed in the small intestine but can be fermented by the microflora in the colon and subsequently exerts beneficial physiological effects. Therefore, RS is regarded as one type of dietary fiber (11). RS can be found in cereal products, seeds, beans, bananas, and potatoes, and in a commercially purified form as an additive to foods (12). The initial development of RS products was mainly based on corn with a high amylose (AM) content, more RS based on cassava, wheat, potato, and barley has also been made with great success (13). RS is classified into five forms, from RS1- to RS5, according to its origin and digestibility, i.e., physically inaccessible starch (RS1), high starch (RS2), modified starch (RS3), chemically modified starch (RS4), and starch-lipid complexes (RS5) (14). The unique properties of RS, such as its natural origin, mild flavor, white color, and low water retention, have made it a prominent topic for research on a variety of functional foods (15).

Based on accumulating evidence, RS is beneficial for human health. Animal studies have shown that RS intake ameliorates intestinal dysbiosis and chronic inflammation (11). Similar results have been obtained from population trials: wheat high in RS modulated fecal metabolites and microorganisms which are associated with gastrointestinal health in healthy adults (16). A meta-analysis showed that RS improved total cholesterol, low-density lipoprotein (LDL) cholesterol, and tumor necrosis factor-α (TNF-α) levels (17). The most well-studied function of RS might be the ability to improve glucose homeostasis. RS intervention has consistently been shown to help control postprandial glucose levels (18, 19). However, most of the published studies used RS as supplement in processed products [such as bagels (20) and cereal bars (21)] which are often consumed as snacks. Rice, on the contrary, as a main staple food for more than half of the global population (22) would be an ideal carrier for RS. Since refined ordinary rice usually has low fiber and high glycemic index (GI), it is not recommended as a daily staple food for patients with diabetes. So, cultivating high-RS rice with hypoglycemic properties may be a good option for diabetes control (23, 24). Although evidence for RS in reducing postprandial glycemic response is well-documented, most studies on diabetes used RS as supplement. Therefore, this study is about to confirm the effect of newly cultured high-RS rice on postprandial blood glucose levels in patients with type 2 diabetes, which may provide scientific evidence for the promotion of high-RS rice consumption to improve the health of diabetes.

## Materials and methods

### Study design

This study was a randomized, double-blinded controlled trial. The study was approved by the Research Ethics Committee of Chongqing Medical University (2021089). Subjects signed informed consent to take part in.

The trial site was in the Health Management Center of the Second Hospital of Chongqing Medical University, Chongqing, China.

### Subjects

Subjects were recruited through the advertisement posted on the official social media (WeChat) account of the Health Management Center of the Second Hospital of Chongqing Medical University between November 2021 and February 2022. People who were interested in this study contacted the researcher for details. Inclusion criteria included: (1) age is between 40–70 years old; (2) diagnosed type 2 diabetes has medical records; (3) the patient's condition is stable without acute complications; (4) having the ability to do social communication. Exclusion criteria included patients with serious diseases or conditions, such as malignant tumors, severe heart disease, stroke, liver and kidney insufficiency, acute respiratory infections, fever, and those who have limited mobility. Subjects were interviewed at the first time of testing for basic information. A total of 106 patients with diagnosed type 2 diabetes finally were recruited.

### Random grouping

Subjects were randomly assigned into two groups according to the order of testing. Group A: RS rice then ordinary rice; Group B: ordinary rice then RS rice.

Cards labeled with group assignments (Group A; Group B) were placed into sealed envelopes. The first time when subjects arrived at the test site, they were given an envelope randomly to decide the test order of the two rices. The rices of testing were blinded (cooked rices were indistinguishable by texture and taste in our pretest) to study subjects and the blood glucose sample testers.

### Preparing testing meals

The Chongqing Academy of Agricultural Sciences cultured and provided both types of refined rice, i.e., high-RS rice and ordinary rice. High-RS rice has 8.44% of resistant starch, while it is only 0.46% in ordinary rice. The nutrition facts were shown in Table 1.

**Table 1:** Nutrition facts of test rices^*^ (per 100 g refined rice).

**Item**	**Unit**	**High-RS rice**	**Ordinary rice**
Energy	KJ	1,485	1,462
Protein	G	7.7	6.6
Fat	G	0.9	0.6
Carbohydrates	G	77.1	77.7
Sodium	Mg	0	0
Dietary fiber	G	1.2	0.8
Resistant starch content	G	8.44	0.46

* Data were reported by an independent qualified laboratory in Beijing, China.

Rice was weighed, washed once with water, and cooked by using an electric rice cooker. The final rice-to-water ratio for cooking was 1:1.8 (decided by the taste and texture of cooked rice in pretest). The cooked rice/raw rice ratio was 2.39 and 2.46 for high-RS rice and ordinary rice, respectively (measured in pretest).

Three sizes of cooked rice (large portion: 200 g cooked rice; medium portion: 150 g cooked rice; small portion: 100 g cooked rice) were provided for subjects to choose according to their appetite.

To simulate the real diet, side dishes were also added, including a portion of boiled bok choy (100 g raw vegetable, 5 ml of soy sauce, and 5 ml of sesame oil), one cup (200 ml) of soup (5 grams nori and 1 stirred egg boiled in 2 liters of water), 1 boiled egg, and a small pinch of pickled vegetable.

Meals were freshly prepared on the testing day morning by the cafeteria staff in the hospital.

### Test procedure

One week before testing day, subjects were required to maintain their usual lifestyle. On the day before the testing, subjects were told to avoid foods high in dietary fiber and sugar at dinner, to fast after 10:00 pm, and to keep fasting until the testing in the next morning. Subjects were also required to bring any medications they were prescribed to take in the morning, including hypoglycemic drugs.

Before taking the testing meal, 1 ml of fasting venous blood was collected to measure the fasting glucose. At their first bite of the meal, it was defined as the zero-time of trial. The whole meal was eaten up in 10 min. After the meal, subjects were reminded to take their medicine if prescribed.

Each time at 30, 60, 120, and 180 min after the meal, 1 ml of venous blood was drawn. Blood samples were collected from the subjects by nurses and sent to the hospital laboratory to do blood glucose tests.

After a 7-days washout period, the second round trial was conducted with the other rice according to the test order assigned at the first trial. In the second round, all except the rice type remained the same as in the first round for each subject, including meal size and medications.

### Outcome indicators

Outcome indicators included the change of postprandial glucose level [ΔCt (mmol/L)] and the incremental area under the glucose curve (IAUC [mmol/ (L·min)]).

ΔCt is blood glucose level at a time point minus fasting blood glucose level.

IAUC is calculated using the trapezoidal rule, with the fasting blood glucose level as the baseline and ignoring the area below the baseline.

### Statistical analysis

All continuous values were expressed as the mean ± standard deviation (SD). To control the confounding factors, studends *t*-tests and chi-square tests were used to analyze the baseline situation of the two AB groups. Paired *t*-test was used to compare the difference between groups at each time point. Repeated-measures ANOVA was used to detect the effect of test order (25). Statistical analysis was performed using SPSS 25.0 software. *P* < 0.05 was considered statistically significant.

## Results

### Overview of subjects

**Figure 1:**
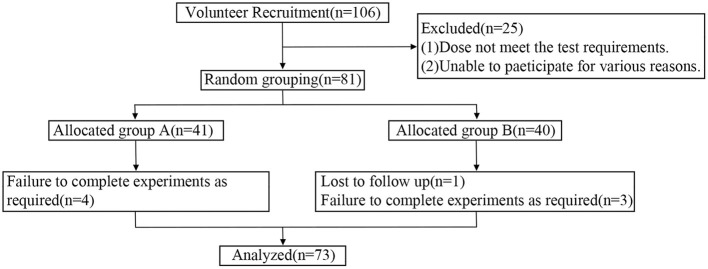
Flow diagram of participant recruitment and included.

Eighty one out of 107 recruited patients with type 2 diabetes eventually were eligible for this study. During testing, 1 subject who failed two rounds of trial and 7 subjects who failed to fully follow the instructions were excluded from the data analysis. Ultimately, 73 subjects were included in this study. The process of subject screening was shown in Figure 1.

For those who were included, the age was 59.74 ± 8.89 years, the body mass index (BMI) was 23.22 ± 2.66 kg/m^2^, and the duration of diabetes was 9.49 ± 7.02 years. There were no significant differences between the two groups (two testing orders) in terms of baseline conditions such as mean age, sex, height, weight, and BMI (*P* > 0.05). The profile of the subjects was shown in Table 2.

**Table 2:** General condition of the subjects (*n* = 73).

**Item**	**Total** ** (*n* = 73)**	**Group A** ** (*n* = 37)**	**Group B** ** (*n* = 36)**	***P***
Age (years)	59.74 ± 8.89	58.14 ± 8.60	61.39 ± 8.99	0.118
Sex (male/female)	46 / 27	25 / 12	21 / 15	0.414
Height (cm)	163.42 ± 7.77	163.35 ± 8.81	163.50 ± 6.65	0.936
Body weight (kg)	62.04 ± 8.27	62.60 ± 8.71	61.46 ± 7.87	0.559
BMI (kg/m^2^)	23.22 ± 2.66	23.45 ± 2.63	22.99 ± 2.71	0.462
Hypertension (with/without)	19 / 54	10 / 27	9 / 27	0.844
Duration of diabetes	9.49 ± 7.02	9.89 ± 6.67	9.08 ± 7.44	0.626
Anti-diabetic drugs (yes/no)	61 / 12	31 / 6	30 / 6	0.959
Other prescribed drug (yes/no)	9 / 64	5 / 32	4 / 32	0.755
Rice portion (small, medium, and large)	0 / 8 / 65	0 / 1 / 36	0 / 7 / 29	0.056

Each value is expressed as the mean ± SD. Group A, RS rice then ordinary rice; Group B, ordinary rice then RS rice.

### The postprandial blood glucose level

The paired *t*-test showed that the subjects when taking the cooked high-RS rice had significantly lower changes in postprandial blood glucose than the time when taking cooked ordinary rice at 30 and 60 min (*P* < 0.05, Table 3).

**Table 3:** Comparisons of postprandial blood glucose levels (mean ± SD) between high-RS rice meal and ordinary rice meal (*n* = 73).

**Item**	**High-RS rice**	**Ordinary rice**	***t***	***P***
ΔC_30min_ (mmol/L)	2.80 ± 1.38^*^	3.04 ± 1.50	−2.057	0.043
ΔC_60min_ (mmol/L)	3.94 ± 2.25^*^	4.25 ± 2.29	−2.131	0.036
ΔC_120min_ (mmol/L)	3.00 ± 2.56	3.14 ± 2.54	−0.698	0.488
ΔC_180min_ (mmol/L)	1.13 ± 2.58	1.16 ± 2.17	−0.158	0.875

ΔCt is the change between postprandial blood sugar and fasting blood glucose.

* P < 0.05 (paired t-test).

### Incremental area of the blood glucose curve

The incremental area under the blood glucose curve in patients with diabetes when taking cooked high-RS rice was smaller than that when taking cooked ordinary rice. The paired *t*-test revealed statistically significant differences between the two kinds of rice at 30, 60, and 120 min (*P* < 0.05, Table 4).

**Table 4:** Comparison of IAUC (mean ± SD) between high-RS rice meal and ordinary rice meal (*n* = 73).

**Item**	**High-RS rice**	**Ordinary rice**	***t***	***P***
IAUC_30min_ [mmol/(L·min)]	42.04 ± 20.65^*^	45.53 ± 22.45	−2.057	0.043
IAUC_60min_ [mmol/(L·min)]	143.54 ± 69.63^*^	155.15 ± 73.53	−2.277	0.026
IAUC_120min_ [mmol/(L·min)]	354.61 ± 191.96^*^	379.78 ± 195.30	−2.066	0.042
IAUC_180min_ [mmol/(L·min)]	493.33 ± 308.03	519.78 ± 304.21	−1.381	0.171

IAUC, incremental area under the postprandial glucose curve.

* P < 0.05 (paired t-test).

### The impact of testing order

Repeated-measures ANOVA showed that the treatment factors (types of rice) but not the test order caused the differences in postprandial blood glucose in patients with diabetes at 30 and 60 min (*P* < 0.05, Table 5).

**Table 5:** Repeated ANOVA of postprandial glucose by test order.

**Item**	**Group A**	**Group B**	**Repeated-measures ANOVA(** ***P)***	

	**(*****n*** = **37)**	**(*****n*** = **36)**	**Treat**	**Test order**
ΔC_30min_ (mmol/L)	2.86 ± 1.50	2.98 ± 1.38	0.045^*^	0.297
ΔC_60min_ (mmol/L)	3.97 ± 2.32	4.22 ± 2.22	0.036^*^	0.085
ΔC_120min_ (mmol/L)	3.04 ± 2.59	3.09 ± 2.50	0.493	0.797
ΔC_180min_ (mmol/L)	1.18 ± 2.33	1.10 ± 2.42	0.871	0.676

Group A, RS rice then ordinary rice; Group B, ordinary rice then RS rice.

* P < 0.05.

## Discussion

As a metabolic disease, diabetes will gradually progress with a variety of acute and chronic complications of varying severities, which is the main cause of disability and death of patients with diabetes (26). The development of diabetic complications is not only associated with persistent chronic hyperglycemia but also closely related to fluctuations in blood glucose levels (27, 28).

We have found in this study that patients with type 2 diabetes when consuming high-RS rice had smaller fluctuations in postprandial blood glucose levels than when consuming ordinary rice in the real world with antidiabetic drugs and simulated ordinary diet, and their glycemic changes were significantly lower at 30 min and 60 min after meals when consuming high-RS rice. Moreover, the incremental area under the blood glucose curve of patients with diabetes when consuming high-RS rice was also smaller than that of patients when consuming ordinary rice.

Other studies have also reported similar results. For example, Yuhi Saito (29) conducted two randomized, single-blinded, crossover trials to study the effects of a single intake of high-RS crackers and cooked high-RS rice on postprandial glycemic and insulin responses in healthy adults and compared them with rice crackers and cooked rice prepared from ordinary varieties, and the results indicated that both high-RS rice crackers and high-RS rice had lower postprandial glycemic and insulin responses. Takahashi (30) conducted a non-randomized crossover design trial in which five healthy men consumed two types of cooked rice, i.e. control (low-RS) and test (high-RS) rice, and found that high-RS rice improved postprandial hyperglycemia and hyperinsulinemia. But unlike our study, those studies investigated postprandial blood glucose in healthy people. The present study showed that naturally cultured high-RS rice has the same effects on postprandial blood glucose in type 2 diabetes. Although the benefits of resistant starch on diabetes have been reported in some studies, nearly all studies used RS supplements as intervention and postprandial blood glucose was not measured (31). Until now, the effects of naturally cultured high-RS rice on diabetes were rarely reported.

RS, as a special dietary carbohydrate, is considered one of the most important factors in the study of the relationship between carbohydrates and health (31). In our study, the refined high-RS rice has 8.44% of resistant starch, obviously high than ordinary refined rice which has <1% of RS. Not surprisingly, studies have shown that high-RS rice has a lower GI value than ordinary rice (32). Numerous studies have shown that the consumption of low-GI food has improved the drastic changes in postprandial blood glucose levels (33, 34). Moreover, a significant negative correlation was also observed between GI and RS (35). The novel high-RS rice in this study have a GI value of 65 when cooked, which is lower than 80 of ordinary cooked rice (result from our preliminary study, data have not been reported). The low GI value may explain the reduced postprandial glycemic response of high RS rice in diabetes compared with ordinary rice.

There are some strengths in this study. Firstly, randomized controlled trial (RCT) design was used in this study which can effectively control kinds of confounders. Secondly, a double-blinding method was used to avoid possible bias related to the researchers and subjects. Thirdly, postprandial glucose was measured at multiple time points, which provided an overall view of high-RS rice on postprandial glucose. Fourthly, this study tested high-RS rice in real-life conditions by simulating the real diet and allowing the medicine prescribed (31). Therefore, the results are reliable and can be generalized to the real world.

Some limitations of our study also must be mentioned. Since this study only measured postprandial glucose, the long term effects of high-RS rice on blood sugar and other health effects are still undecided. Therefore, studies about high-RS rice on diabetes or other metabolic diseases in the long term with more biomarkers are warranted.

## Conclusion

The novel high-RS rice as a staple food when substituting for widely consumed ordinary rice may provide potential health benefits by lowering blood glucose in patients with type 2 diabetes.

## Data availability statement

The raw data supporting the conclusions of this article will be made available by the authors, without undue reservation.

## Ethics statement

The studies involving human participants were reviewed and approved by Ethics Committee of Chongqing Medical University. The patients/participants provided their written informed consent to participate in this study approval number: 2021089.

## Author contributions

YZ and L-LT reviewed the literature and completed the protocol design. L-LT, YM, X-YQ, W-QD, and M-XC conducted the clinical trial and were responsible for data collection. L-LT performed the statistical analysis and data interpretation under the direction of YZ, wrote the manuscript, and revised the manuscript. All authors read and approved the final manuscript.
